# Antimicrobial resistance profiles of bacterial pathogens from feline urinary tract infections in Rio de Janeiro, Brazil

**DOI:** 10.1007/s11259-026-11415-w

**Published:** 2026-07-23

**Authors:** Barbara Barreto Oliveira, Camila Alves Maia da Silva, Izabella Brito de Souza, Milena Gomes Cabral, Bianca Costa Silva, Beatriz Rodrigues Pellegrina Soares, Juliana Silva do Nascimento, Thais Abrantes Rodrigues, Fulvio Martins Ambrosio Aquino, Waldemir Silva de Aguiar, Mariana Severo Ramundo

**Affiliations:** 1 Departamento de Clínica Médica, Disciplina de Imunologia Clínica e Alergia, Faculdade de Medicina da Universidade de São Paulo, Av. Dr. Enéas de Carvalho Aguiar, 44, INCOR 9º andar, Bloco 02, Cerqueira César, São Paulo, SP CEP 01246-903 Brazil; 2 Alpha Science, Grupo Alpha, Rio de Janeiro, Brazil

**Keywords:** Feline urinary tract infection, Antimicrobial resistance, Multidrug resistance, Antimicrobial susceptibility, One health

## Abstract

This study characterized the bacterial epidemiology and antimicrobial resistance profiles of 448 isolates recovered from feline urine cultures in Rio de Janeiro, Brazil, between 2020 and 2024. Antimicrobial susceptibility was assessed by disk diffusion and interpreted according to CLSI VET01S and Brazilian Committee on Antimicrobial Susceptibility Testing (BrCAST) breakpoints. *Escherichia coli* was the predominant pathogen (45.1%), followed by *Proteus* spp. (16.3%) and *Klebsiella* spp. (12.5%). Resistance to antimicrobials recommended as first-line options by the ISCAID guidelines was frequent among *E. coli* isolates, including trimethoprim-sulfamethoxazole (57.2%), cephalexin (52.5%), and amoxicillin-clavulanate (43.9%). Veterinary fluoroquinolones showed moderate resistance (enrofloxacin 28.5%, marbofloxacin 20.9%), while nitrofurantoin resistance reached 37.7%. Third-generation cephalosporins and carbapenems retained the highest activity (ceftazidime 13.2%; meropenem 0%). Multidrug resistance (MDR) was observed in 55.8% of all isolates, with the highest prevalence in *Proteus* spp. (79.5%) and *E. coli* (57.9%). Resistance to third-generation cephalosporins was detected in 22.8% of *E. coli* isolates, and carbapenem resistance was detected in 5 isolates from different species. These findings indicate that resistance to first-line agents is common in feline uropathogens in this setting and support the routine use of urine culture and antibiotic susceptibility testing to guide antimicrobial therapy.

## Introduction

Urinary tract infections (UTIs) are among the most frequent indications for antimicrobial therapy in feline medicine, especially in older cats and in those with concurrent conditions such as chronic kidney disease or diabetes mellitus (Weese et al. 2019; Dorsch et al. 2019). According to the guidelines published by the International Society for Companion Animal Infectious Diseases (ISCAID), amoxicillin and trimethoprim-sulfamethoxazole are considered first-line options for sporadic bacterial cystitis, and urine culture with susceptibility testing is recommended in all cats with suspected bacterial UTI (Weese et al. 2019). *Escherichia coli* is the most commonly isolated uropathogen in cats worldwide, followed by species of *Proteus*, *Klebsiella*, *Staphylococcus*, and *Enterococcus* (Dorsch et al. 2015; Temmerman et al. 2024; Hernando et al. 2021). However, growing resistance to commonly used antimicrobials, together with the emergence of multidrug-resistant (MDR) and extended-spectrum beta-lactamase-producing strains (ESBL), has raised concerns about the effectiveness of empirical protocols and the role of companion animals as reservoirs of resistance within the household environment (Marques et al. 2018; Aurich et al. 2022). In Brazil, published data on antimicrobial resistance in feline urinary pathogens are scarce, and laboratory-based surveillance studies focused specifically on cats are lacking for most major urban centers. Rio de Janeiro, one of the largest cities in Brazil, currently has no published species-specific resistance data for feline uropathogens based on antimicrobials with established veterinary indications. The present study aimed to describe the distribution of bacterial species, antimicrobial resistance profiles, and the prevalence of clinically relevant resistance markers in isolates recovered from feline urine cultures over a five-year period in Rio de Janeiro.

## Materials and methods

This retrospective study was based on a secondary analysis of routine diagnostic records from feline urine cultures processed between January 2020 and December 2024 at Alpha Science/Grupo Alpha, a veterinary diagnostic laboratory in Rio de Janeiro. No additional sample collection or laboratory processing was performed for the purposes of this study. Bacterial identification was performed using conventional microbiological methods. Antimicrobial susceptibility testing was performed using the disk diffusion method. Results were interpreted according to the Clinical and Laboratory Standards Institute veterinary breakpoints (CLSI VET01S; CLSI 2024) and the Brazilian Committee on Antimicrobial Susceptibility Testing (BrCAST) criteria and recorded as susceptible (S) or resistant (R); intermediate results were excluded from resistance calculations. Each isolate was treated as an independent observation.

Antimicrobial agents were classified into pharmacological groups, and only agents with clinical indications for UTI treatment and veterinary regulatory approval were included in the resistance analyses, in accordance with the ISCAID guidelines (Weese et al. 2019) and the CLSI VET01S standard (CLSI 2024). Tetracyclines were excluded because they are not part of the recommended susceptibility panel for urinary Enterobacterales, and ciprofloxacin, norfloxacin, and levofloxacin were excluded because they lack veterinary approval for systemic use in companion animals. Resistance rates were calculated as the proportion of resistant isolates among those tested for each agent, excluding agents tested in fewer than 15 isolates per species. MDR was defined as resistance to three or more antimicrobial classes. Resistance to third-generation cephalosporins, fluoroquinolones, and carbapenems was evaluated as an indicator of clinically important resistance. All analyses were conducted in R (version 4.4). This study used anonymized retrospective data, and formal ethical approval was not required under institutional guidelines.

## Results

The 448 isolates were recovered from 396 individual cats; 33 animals contributed more than one isolate from separate urine cultures submitted on different dates. Among these, 9 animals (27.3%) yielded the same bacterial species across all cultures, while 24 (72.7%) had different species recovered in subsequent samples. The median interval between consecutive cultures was 78 days (IQR, 42 to 133), suggesting that most repeat cultures represented distinct infectious episodes rather than follow-up of the same infection. The number of cultures varied across years (17 in 2020, 142 in 2021, 139 in 2022, 117 in 2023, and 33 in 2024), reflecting changes in sample submission volume over the study period, which included the early phase of the COVID-19 pandemic. Males accounted for 270 (60.3%) and females for 174 (38.8%) of the animals, with four records lacking sex information. The median age was 5.0 years (IQR, 3.6–10.0).

*Escherichia coli* was the most frequently isolated species, accounting for 202 of 448 isolates (45.1%), followed by *Proteus* spp. (73; 16.3%), *Klebsiella* spp. (56; 12.5%), staphylococci other than *S. aureus* (SOSA; 40; 8.9%), and *Enterococcus* spp. (21; 4.7%). The complete species distribution is shown in Table 1.

**Table 1:** Distribution of bacterial species isolated from 448 feline urine cultures in Rio de Janeiro, Brazil (2020 to 2024)

Bacterial species	*n*	%
*Escherichia coli*	202	45.1%
*Proteus spp.*	73	16.3%
*Klebsiella spp.*	56	12.5%
*Staphylococci* other than *S. aureus* (SOSA)	40	8.9%
*Enterococcus spp.*	21	4.7%
*Non-fermenting Gram-negative bacilli*	15	3.3%
*Other Staphylococcus spp.*	13	2.9%
*Coagulase-positive Staphylococcus*	8	1.8%
*Streptococcus spp.*	5	1.1%
*Others*	15	3.3%

**Fig. 1:**
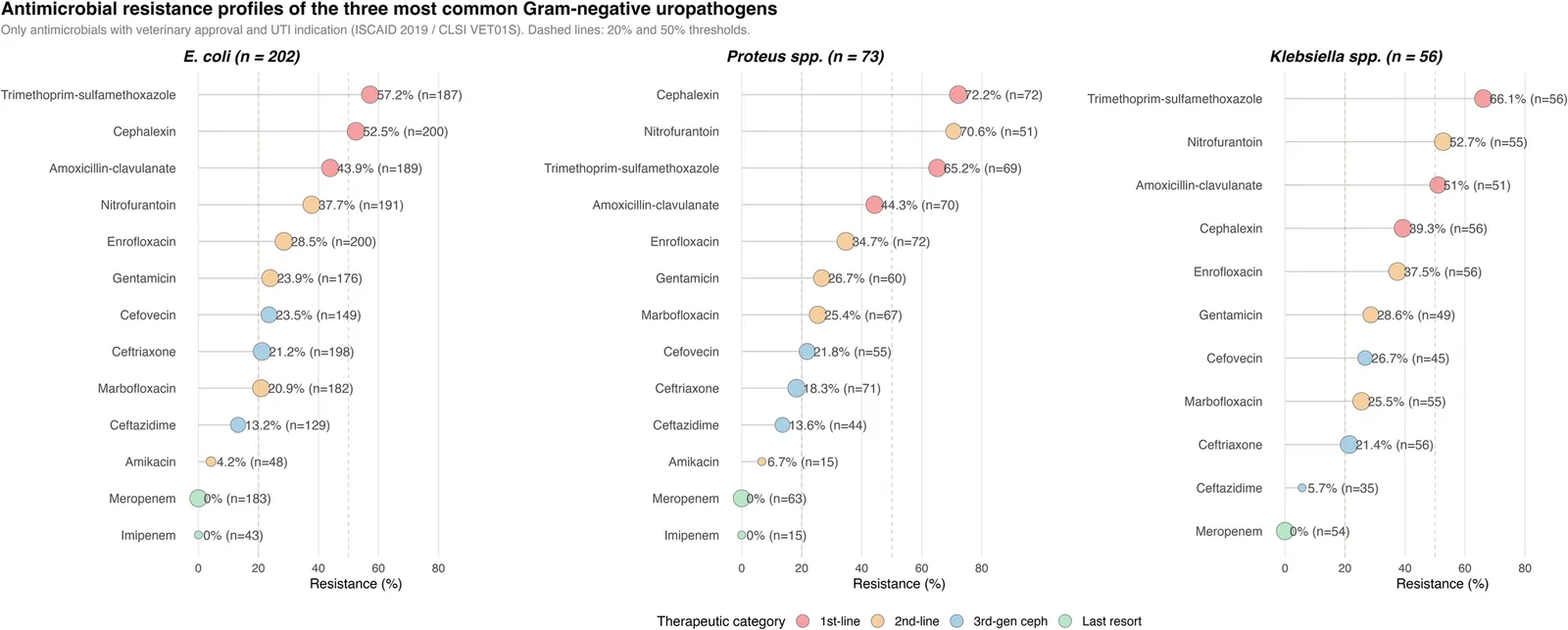
Antimicrobial resistance profiles of the three most common Gram-negative uropathogens isolated from feline urine cultures. Only antimicrobials with veterinary approval and clinical indication for urinary tract infections are shown, in accordance with the ISCAID (2019) guidelines and the CLSI VET01S (2024) criteria. Resistance rates were calculated over the number of isolates effectively tested for each agent. Dashed lines indicate 20% and 50% resistance thresholds. Bubble size reflects the number of isolates tested. Therapeutic categories: first line (pink), second line (orange), third-generation cephalosporin (blue), and last resort (green)

Among *E. coli* isolates, resistance to the first-line agents recommended by the ISCAID guidelines was high: trimethoprim-sulfamethoxazole 57.2% (107/187 tested), cephalexin 52.5% (105/200), and amoxicillin-clavulanate 43.9% (83/189). Nitrofurantoin resistance reached 37.7% (72/191). Resistance to veterinary fluoroquinolones was moderate, with rates of 28.5% for enrofloxacin (57/200) and 20.9% for marbofloxacin (38/182). Gentamicin resistance was 23.9% (42/176). Third-generation cephalosporins showed lower rates of resistance: ceftriaxone, 21.2% (42/198); ceftazidime, 13.2% (17/129). Carbapenems retained full activity, with no resistance detected among *E. coli* isolates (0/183 for meropenem; 0/43 for imipenem). Resistance profiles for *E. coli*, *Proteus* spp., and *Klebsiella* spp. are presented in Fig. 1.

Overall, 250 of 448 isolates (55.8%) met the criteria for MDR. The highest MDR prevalence was observed in *Proteus* spp. (58/73; 79.5%), followed by *Klebsiella* spp. (34/56; 60.7%) and *E. coli* (117/202; 57.9%). MDR was less common in SOSA (15/40; 37.5%) and *Enterococcus* spp. (4/21; 19.0%). Resistance to third-generation cephalosporins was detected in 22.8% of *E. coli*, 23.3% of *Proteus* spp., and 21.4% of *Klebsiella* spp. Fluoroquinolone resistance ranged from 22.5% in SOSA to 44.6% in *Klebsiella* spp. Five isolates of different species showed carbapenem resistance, including *Acinetobacter baumannii*, *Morganella morganii*, *Enterococcus* spp., *Staphylococcus aureus*, and SOSA. Among *E. coli* isolates tested with both ceftriaxone and cefoxitin, 12 showed a phenotypic pattern consistent with ESBL production (ceftriaxone-resistant, cefoxitin-susceptible). Resistance markers for the five most common species are summarized in Table 2.

**Table 2:** Antimicrobial resistance markers among the five most common bacterial species from feline urine cultures

Bacterial species	*n*	MDR	Cef3G-*R*	FQ-*R*	Carba-*R*
*Escherichia coli*	202	117 (57.9%)	46 (22.8%)	64 (31.7%)	0 (0%)
*Proteus spp.*	73	58 (79.5%)	17 (23.3%)	26 (35.6%)	0 (0%)
*Klebsiella spp.*	56	34 (60.7%)	12 (21.4%)	25 (44.6%)	0 (0%)
*SOSA*	40	15 (37.5%)	5 (12.5%)	9 (22.5%)	1 (2.5%)
*Enterococcus spp.*	21	4 (19.0%)	0 (0%)	7 (33.3%)	1 (4.8%)

*MDR* multidrug resistance (resistance to three or more antimicrobial classes), *Cef3G-R* third-generation cephalosporin resistance, *FQ-R* fluoroquinolone resistance, *Carba-R* carbapenem resistance, *SOSA* staphylococci other than *S. aureus*

## Discussion

This study describes the antimicrobial resistance profiles of bacterial pathogens isolated from feline urine cultures in Rio de Janeiro, restricting the analysis to agents with established clinical indications and veterinary approval for UTI treatment. The finding that *E. coli* accounted for 45.1% of isolates is in agreement with surveillance data from Europe (Dorsch et al. 2015; Temmerman et al. 2024), Hong Kong (Chan et al. 2022), Spain (Hernando et al. 2021), and Thailand (Lapcharoen et al. 2025). However, the relative frequency of *Proteus* spp. and *Klebsiella* spp. as secondary pathogens was higher than in European studies, where Gram-positive organisms are more commonly reported as the second most common (Dorsch et al. 2015; Aurich et al. 2022).

The observed rates of resistance to first-line antimicrobials are a cause for concern. In our dataset, trimethoprim-sulfamethoxazole and cephalexin exceeded 50% resistance among *E. coli* isolates, and amoxicillin-clavulanate approached 44%. These figures are considerably higher than those reported in European surveillance programs, where resistance to most first-line agents remained below 10% in feline *E. coli* (Temmerman et al. 2024), and are also higher than the rates reported in Southern Europe (Rampacci et al. 2018). Similarly, high resistance levels have been reported in Thailand (Lapcharoen et al. 2025) and in a Polish study on canine and feline urinary *E. coli* (Janczak et al. 2024), suggesting that the pattern we observed is not unique to Brazil but is shared across regions with comparable antimicrobial prescribing practices. These data challenge the direct application of ISCAID empirical recommendations without accounting for local resistance patterns (Weese et al. 2019; Johnstone 2020).

Nitrofurantoin, which is often cited as a favorable empirical option for uncomplicated lower UTIs (Vercelli et al. 2023; Maaland and Guardabassi 2011), showed 37.7% resistance among *E. coli* isolates in our population. This is substantially higher than the near-universal susceptibility described in European and North American studies and suggests that local susceptibility data should be considered before prescribing this agent. According to the ISCAID guidelines, nitrofurantoin is recommended as an alternative for sporadic cystitis caused by susceptible organisms, while fluoroquinolones and third-generation cephalosporins should be reserved for cases in which first-line agents (amoxicillin-clavulanate and trimethoprim-sulfamethoxazole) are documented as resistant (Weese et al. 2019). Third-generation cephalosporins and carbapenems retained the highest activity, with ceftazidime resistance at 13.2% and no carbapenem resistance among *E. coli*. The detection of 12 *E. coli* isolates with a phenotypic pattern suggestive of ESBL production and five carbapenem-resistant isolates of various species, including *A. baumannii*, is relevant from a One Health perspective, given the proximity between companion animals and their owners in urban domestic settings (Medeiros et al. 2025; Horodyska et al. 2025).

The substantial variation in annual sample volume (ranging from 17 cultures in 2020 to 142 in 2021) should be considered when interpreting any temporal patterns in these data. The low volume in 2020 coincided with the initial phase of the COVID-19 pandemic, when veterinary services in Brazil were severely restricted. Under these conditions, the sampled population was likely enriched for critically ill or treatment-refractory animals, inflating resistance estimates for that period (Tomczyk et al. 2021; Jolley et al. 2023). For this reason, we regard the species-specific resistance profiles, rather than year-to-year comparisons, as the most informative output of this study.

This study has limitations inherent to its retrospective design, including the absence of clinical data on comorbidities, urine collection method, and prior antimicrobial exposure. Although susceptibility testing followed CLSI VET01S and BrCAST standards throughout the study period, revisions in breakpoint editions may have influenced resistance classification for specific agents, particularly enrofloxacin and marbofloxacin, for which susceptibility breakpoints were lowered in recent updates, and amoxicillin-clavulanate, for which species-specific urinary breakpoints were introduced for cats (CLSI 2024). In some cases, the inclusion of multiple isolates from the same animal may have introduced non-independence, although 88.4% of isolates corresponded to unique animals. Despite originating from a single laboratory, the samples were submitted by multiple veterinary clinics across different areas of Rio de Janeiro, one of the largest cities in Brazil. Beyond their clinical relevance for guiding therapy in individual patients, these findings carry broader public health implications. Continuous local monitoring of antimicrobial resistance in companion animals helps identify clinically important resistant organisms, including members of the ESKAPE group, that may circulate between animals and humans in the domestic environment. These data represent the first species-specific resistance profiles available for feline uropathogens in this setting and support the routine use of urine culture and susceptibility testing to guide antimicrobial therapy in cats.

## Data Availability

No datasets were generated or analysed during the current study.
